# Design and Implementation of a New Training Flight Simulator System

**DOI:** 10.3390/s22207933

**Published:** 2022-10-18

**Authors:** Ming-Yen Wei, Shen-An Fang, Ji-Wei Liu

**Affiliations:** Aeronautical System Research Division, Simulation System Section, National Chung-Shan Institute of Science and Technology, Taichung 407, Taiwan

**Keywords:** flight simulator, multi-axis control, motion platform, FlightGear, measurement system

## Abstract

Aircraft flight simulators have good cost efficiency, high reusability, and high flight safety. All airlines and aircraft manufacturing companies choose it as sophisticated training equipment for ground simulation, effectively reducing pilot training costs, ensuring personnel safety and aircraft wear and tear. The new simulator proposed in this paper combines a digital motion-cueing algorithm, flight software and motion platform to make pilots feel as if they are in the real world. By using EtherCAT technology to drive the motion-cueing platform, it can improve the data transmission speed of the simulator as well as the strong anti-interference ability of communication and the control operation efficiency. Therefore, the simulated flight subjects can perform long-distance and large-angle training. Next, a set of measurement systems was established to provide monitoring items including attitude, velocity and acceleration, which can be displayed on the screen and recorded on the computer in real time and dynamically. Finally, seven training subjects were implemented to demonstrate the feasibility and correctness of the proposed method.

## 1. Introduction

Due to the improvement of modern flight technology, with the increasing number of aircraft for various purposes, it is more and more difficult for pilots to train with actual aircrafts, and there is a certain danger in actual aircraft flight training. Therefore, flight simulation [1] provides a good solution for training flight pilots. The flight scenes required by the flight software to produce the visual effect, the realistic scenery and the realistic aircraft dynamics, and the shock of sound and light, provide people with an immersive flight experience. Ground flight simulation can not only save the cost of real aircraft flight, but also ensure the safety of humans and machines, and achieve the purpose of training pilots [2].

Nowadays, most of the flight training uses the Stewart platform [3]. After driving six electric cylinders to achieve six degrees of freedom motion space, the earliest prototype of the Stewart platform appeared in 1956, and was established by V. E. Gough due to the needs of work. [4], The platform was subsequently improved by D. Stewart in 1965, who proposed a parallel six-axis robot in 1965, also known as the Stewart platform [5]. At that time, the Stewart platform was only used to make flight simulators, so the Stewart platform was initially regarded as a standard institution for flight simulators. The Stewart platform is a six-degrees-of-freedom parallel mechanism platform. Since the kinematics and dynamics analysis of the six degree-of-freedom parallel mechanism is very difficult, it was not until 1993 that research team of the French National Academy of Sciences used the geometric analysis method [6], which makes the research of the Stewart platform have a great breakthrough and diversified development. Then application of the six-degrees-of-freedom parallel platform mechanism is also increasing, such as multi-axis processing machine [7], active shock-proof platform [8], medical surgery auxiliary platform system [9], virtual motion simulator [10], underwater vehicle simulation platform [11] and other applications. The structure of the Stewart platform is composed of a fixed bottom plate and a movable upper plate. Through six electric cylinders with variable rod lengths, the parallel motion mechanism formed by the universal joint and the fixed bottom plate is connected with the movable plate respectively, and then coupled by the motor. The change signal of the rod length returned by the optical encoder will make the movable plate move with six degrees of freedom. Since the movable plate of this mechanism is constrained in parallel and the length of six shafts, the movement of six degrees of freedom in space is limited. Since the change of the length of each axis is independently controlled within the range of the movement space of the mechanism, most of them are controlled by a SISO system, and the motors of the respective drive shafts are independently controlled by the rod length [12]. However, the overall motion space is limited and needs to be maintained in a co-planar plane at all times, so long-distance and large-angle motion cannot be provided by the control system.

During a flight mission, the pilot’s visual observation of the indicators and the feel of the aircraft’s movement provide the pilot with perceptible information that enables the pilot to respond appropriately. When the aircraft is in flight, the change of gravity will affect the motion sensor of the pilot’s vestibular system, thereby affecting the pilot’s vision and sense of orientation, and even the control of the aircraft. Therefore, it is necessary to execute the wash-out algorithm for the balance organ of the human body by means of a simulator. Working principle using the inner ear of the vestibular system to perceive linear and rotational speed and acceleration, a sensitive range of motion is displayed on the motion platform, simulating the actual dynamic effect [13]. In addition, in [14], an adaptive control technique is proposed-integrated motion cueing algorithm to improve the control of six servo synchronous tracking, which can perform adaptive parameter estimation within a limited interval to control the amount of control required by the platform. In [15], the model of the filter is designed by predictive control, as the filter needs to modify the parameters each time. In [16], a filter model was designed using the optimization theory to find the optimal parameters via a recursive genetic algorithm. In addition, some scholars use the motion platform to simulate the actual vehicle motion, and construct the realistic road vibration and surge acceleration during the motion of the vehicle, so as to achieve the human body effect of simulator training [17]. In [18], a fuzzy control rule base is established for the rate limiter of the algorithm, and a rate limiter that can be dynamically adjusted on-the-fly is designed by using the specific force error. In [19] mentioned that the discussion about the impact of simulator motion on pilot training has been going on for a long time. In order to prove the difference between static simulator and dynamic simulator, the authors collects various data and uses the meta-analysis method statistical method records 11 evaluation items. Through the statistical results, the two pilots’ learning conditions in the static simulator and the dynamic simulator can be obtained. The dynamic simulator can achieve a better learning effect. In [20] proposed to use the average and standard deviation of the training data of 10 F16 pilots in two situations to find out which pilots need to be trained, indicating that the use of simulators as a training method is not only safe, but also in terms of training costs is also relatively cheap. In the experiment [21], pilots with professional licenses used the simulator CKAS Motion Sim 5 to fly 12 different scenarios to measure the energy consumption of the simulator. Implementing training can reduce the energy consumption by 97%, so it is concluded from the experimental results that the use of simulators can greatly reduce the cost of pilot training.

This paper proposes a six-axis 360-degree motion platform, which was designed with a digital controller to improve the fidelity of dynamic simulation of linear acceleration. The difference with the relative literature was the mechanism design, control architecture, drive method, and image effect. The virtual space synchronous multi-axis motion device for flight simulation was designed and developed in this research. With the IG visual effect map of the flight simulator scene, the real-time dynamic visual effect map is matched with the relevant functions of the platform operation test, allowing the trainer to experience the realistic comparative force and rotation angular velocity [22]. Under this goal, the design can perform long-distance stroke, continuous rotation, other related actions and the technology of the simulation system will be more advanced, more realistic and meet the training needs and other directions.

To design a set of flight simulators, combined with FlightGear [23], a self-developed and self-made platform, motion-cueing algorithm, dual controllers (digital signal processor, DSP and programmable logic controller, PLC) and multi-axis servo drives were used. After receiving the instruction of the control handle, the visual effect computer converts the control parameters into the control data of the image generator, and then returns the linear acceleration and angular velocity information to the digital signal processing. In addition, some sensors were installed at the middle of platform to acquire motion status to track and monitor the performance evaluation. To the best of our knowledge, the ideas we mention have not been presented in previously published papers [1,2,3,4,5,6,7,8,9,10,11,12,13,14,15,16,17,18,19,20,21,22,23]. As a result, this paper presents some new ideas in terms of kinematics and a motion-cueing algorithm, the servo motion is executed by the programmable controller, and the commands are read and received through the Ether-CAT communication port to achieve synchronous simulation. Between the platform and the visual effect content to realize the development of the remote monitoring 6-DoF system and improve the training effect of the pilots.

## 2. The Six-Axis Motion Platform Control

**Figure 1:**
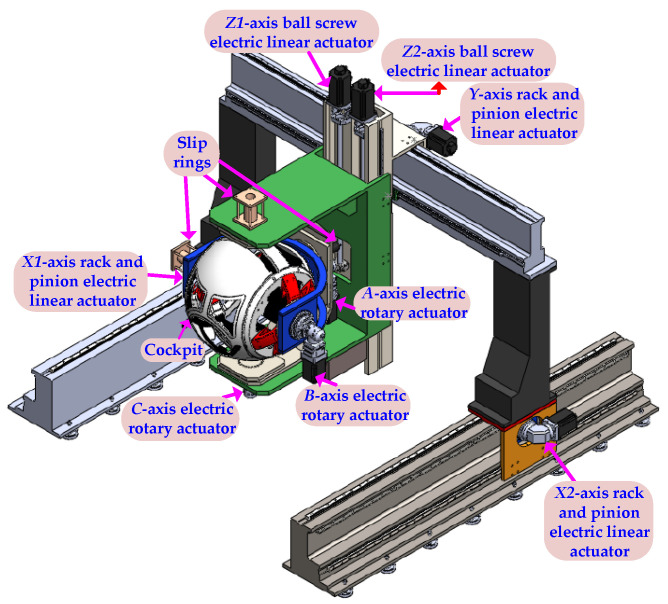
Six degrees of freedom motion platform.

**Figure 2:**
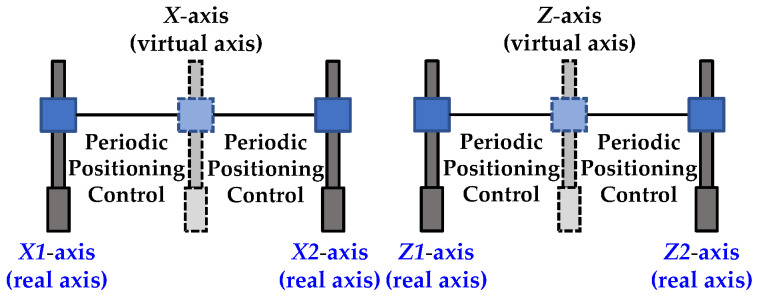
The relationship between the axes and the virtual axis.

The motion-cueing algorithm in this paper adopts the digital optimal tracking motion algorithm design [24]. Figure 1 shows that the surge axis (*X*-axis) and heave axis (*Z*-axis) are in the gantry control mode. The PLC controller uses two virtual axis commands to make the surge1 axis (*X*1), the surge2 (*X*2) real axes and the heave1 axis (*Z*1), heave2 axis (*Z*2) real axes can follow their respective virtual axes to perform motion control, the virtual axis of *X*-axis or *Z*-axis every 1ms cycle time with *X*1-axis or *Z*1-axis, and *X*2-axis or *Z*2-axis moves, as shown in Figure 2.

**Figure 3:**
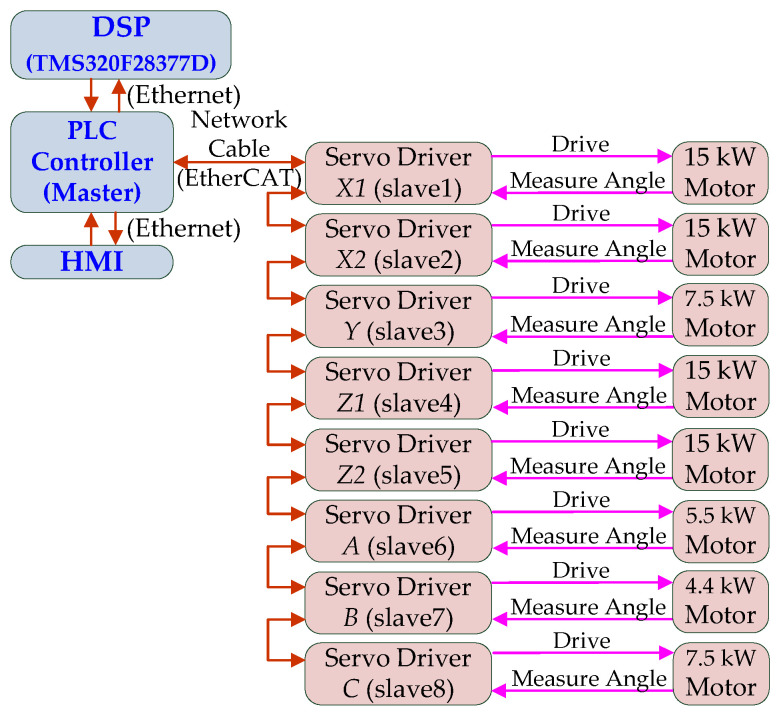
Multi-axis motion control architecture diagram.

The control system uses a PLC controller to perform motion control, and is equipped with a digital motion-cueing algorithm executed by a DSP digital signal processor as the main control terminal, and serial communication (RS-485) and DSP execution data are connected in series. Therefore, in addition to the EtherCAT network communication, the PLC controller can also receive the relevant commands from the DSP through Ethernet, and connect the PLC-EtherCAT communication port to the *X*1, *X*2-axes, Servo drives such as *Y*-axis, *Z*1-axis, *Z*2-axis, *A*-axis, *B*-axis, and *C*-axis, as shown in Figure 3. The 12-inch human-machine interface (HMI) displays the current status of the equipment, the position of each axis, speed, torque and other information.

As shown in the relationship in Figure 3, the PLC controller will read the angles on both sides of (*X*1 and *X*2 real axes) or (*Z*1 and *Z*2 real axes) through EtherCAT, and calculate the center position of the *X* and *Z* real axes, as shown below.

(1) $$\theta_{X}=\frac{\theta_{X1}+\theta_{X2}}{2},\;\theta_{Z}=\frac{\theta_{Z1}+\theta_{Z2}}{2}$$

Therefore, when the virtual axis command is issued, the axis angles of *X*1 and *X*2 and the axis angles of *Z*1 and *Z*2 can be moved to the center position by Equation (1) to follow the virtual axis command. If it is greater than the set 0.1°, the servo motor will stop running immediately. Since the simultaneous motion and stop is based on the servo maximum speed command, maximum acceleration command and maximum deceleration command, the transient speed, acceleration and deceleration commands of each axis are found. The movement amount of *X*-axis, sway axis (*Y*-axis), *Z*-axis, roll axis (*A*-axis), pitch axis (*B*-axis) or yaw axis (*C*-axis) trajectory is used as the reference of motion, then the speed command of six-axis linear interpolation as

(2) $$Vel_{i}=\frac{\left(Vel_{\max}\right)\cdot \left(Disp_{i}\right)}{\left(Disp\right)},\;i=X,Y,Z,A,B,C$$

Similarly, the six-axis acceleration and deceleration commands are

(3) $$Acc_{i}=\frac{\left(Acc_{\max}\right)\cdot \left(Disp_{i}\right)}{\left(Disp\right)},\;i=X,Y,Z,A,B,C$$

(4) $$Dec_{i}=\frac{\left(Dec_{\max}\right)\cdot \left(Disp_{i}\right)}{\left(Disp\right)},\;i=X,Y,Z,A,B,C$$

where

(5) $$Disp=\sqrt{Disp_{X}^{2}+Disp_{Y}^{2}+Disp_{Z}^{2}+Disp_{A}^{2}+Disp_{B}^{2}+Disp_{C}^{2}}$$

Therefore, the relational expressions of speed, acceleration and deceleration under multi-axis drive can be obtained from Equations (2)–(4).

## 3. Kinematics Design

**Figure 4:**
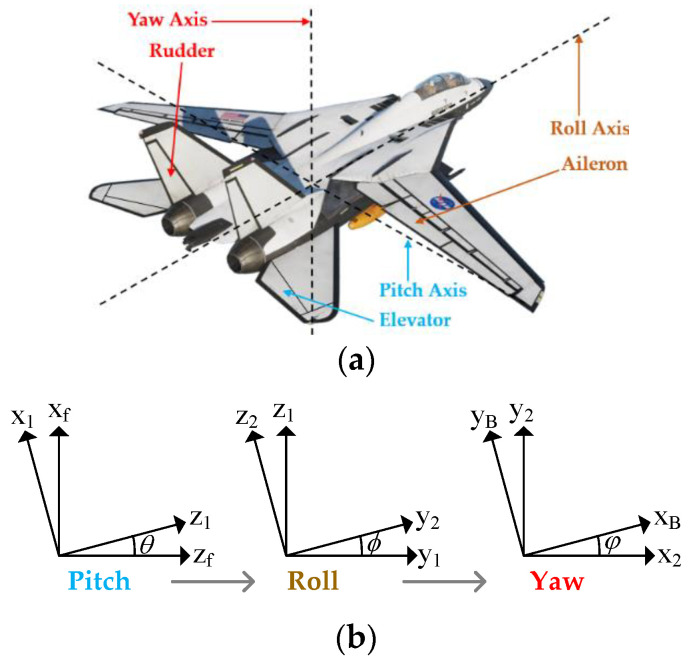
Rotation coordinates: (**a**) flight controls; and (**b**) relationship.

The real six-degrees-of-freedom somatosensory is shown in Figure 1. Therefore, the spherical cockpit of the platform, the inner frame and the outer frame form three rotation axes, and the three linear axes are used to generate the *X*-axis, the *Y*-axis and the *Z*-axis. *A*-xis linear motion effect. Figure 4a shows the relationship between the rudder, aileron, elevator and the three rotation axes of the aircraft, and Figure 4b shows the relationship between the cockpit rotation from the inside to the outside, the order is pitch, roll and yaw, which represents the symbol for θ,ϕ,φ.

The coordinate conversion relationship is obtained as

(6) $$\left[\begin{array}{c} x_{f}\\ y_{f}\\ z_{f} \end{array}\right]=\left[\begin{array}{ccc} \left(\cos\theta \right) & 0 & \left(\sin\theta \right)\\ 0 & 1 & 0\\ -\left(\sin\theta \right) & 0 & \left(\cos\theta \right) \end{array}\right]\left[\begin{array}{c} x_{1}\\ y_{1}\\ z_{1} \end{array}\right]$$

(7) $$\left[\begin{array}{c} x_{1}\\ y_{1}\\ z_{1} \end{array}\right]=\left[\begin{array}{ccc} 1 & 0 & 0\\ 0 & \left(\cos\phi \right) & -\left(\sin\phi \right)\\ 0 & \left(\sin\phi \right) & \left(\cos\phi \right) \end{array}\right]\left[\begin{array}{c} x_{2}\\ y_{2}\\ z_{2} \end{array}\right]$$

(8) $$\left[\begin{array}{c} x_{2}\\ y_{2}\\ z_{2} \end{array}\right]=\left[\begin{array}{ccc} \left(\cos\psi \right) & -\left(\sin\psi \right) & 0\\ \left(\sin\psi \right) & \left(\cos\psi \right) & 0\\ 0 & 0 & 1 \end{array}\right]\left[\begin{array}{c} x_{B}\\ y_{B}\\ z_{B} \end{array}\right]$$

From the conversion relationship shown in (11)–(13), the relationship between the inertial coordinate vector $V^{I}$ and the $V^{B}$ body coordinate vector can be sorted out

(9) VI=RIBVB

where: RIB is the rotation matrix from body coordinates to inertial coordinates can be written as

(10) RIB=RyRxRz

where $R_{y}$, $R_{x}$, and $R_{z}$ are the rotation matrices for the *Y*, *X*, and *Z*-axes, respectively. Substitute the above formula into (11)–(13), and get

(11) RIB=[(cosθ)0(sinθ)010−(sinθ)0(cosθ)][1000(cosϕ)−(sinϕ)0(sinϕ)(cosϕ)][(cosψ)−(sinψ)0(sinψ)(cosψ)0001]=[(cosψcosθ)+(sinϕsinψsinθ)(cosψsinϕsinθ)−(cosθsinψ)(cosϕsinθ)(cosϕsinφ)(cosϕcosψ)−(sinϕ)(cosθsinϕsinψ)−(cosψsinθ)(sinφsinθ+cosφcosθsinϕ)(cosϕcosθ)]

The inertial coordinate transformation matrix TIB is

(12) TIB=[(cosψcosθ)+(sinϕsinψsinθ)(cosψsinϕsinθ)−(cosθsinψ)(cosϕsinθ)x(cosϕsinψ)(cosϕcosψ)−(sinϕ)y(cosθsinϕsinψ)−(cosψsinθ)(sinψsinθ)+(cosψcosθsinϕ)(cosϕcosθ)z0001]=[nxoxaxpxnyoyaypynzozazpz0001]

The angular velocity of the simulator body coordinate rotation is expressed as

(13) $${\vec{\omega }}^{B}={\left[\begin{array}{ccc} p & q & r \end{array}\right]}^{T}$$

The angular velocity of the simulator axis coordinate rotation is written as

(14) $$\dot{\Phi }={\left[\begin{array}{ccc} \dot{\phi } & \dot{\theta } & \dot{\psi } \end{array}\right]}^{T}$$

Arranged from Equations (13) and (14) and Figure 4b, one can obtain

(15) $$p\left({\vec{x}}_{B}\right)+q\left({\vec{y}}_{B}\right)+r\left({\vec{z}}_{B}\right)=\dot{\theta }\left({\vec{y}}_{1}\right)+\dot{\phi }\left({\vec{x}}_{2}\right)+\dot{\psi }\left({\vec{z}}_{B}\right)$$

Arrange (7) and (8) into

(16) $$\begin{array}{l} {\vec{y}}_{1}=\left(\cos\phi \right){\vec{y}}_{2}-\left(\sin\phi \right){\vec{z}}_{2}\\ \;\;\;\;\;\;=\left(\cos\phi \sin\psi \right){\vec{x}}_{B}+\left(\cos\phi \cos\psi \right){\vec{y}}_{B}-\sin\phi {\vec{z}}_{B} \end{array}$$

Substituting Equations (16) and (8) into Equation (15), as follows

(17) $$\begin{array}{l} p\left({\vec{x}}_{B}\right)+q\left({\vec{y}}_{B}\right)+r\left({\vec{z}}_{B}\right)\\ =\left(\dot{\theta }\cos\phi \cos\psi +\dot{\phi }\cos\psi \right){\vec{x}}_{B}+\left(\dot{\theta }\cos\phi \cos\psi -\dot{\phi }\sin\psi \right)\\ \;\;\;\;\;\;+\left(\dot{\psi }-\dot{\theta }\sin\phi \right){\vec{z}}_{B} \end{array}$$

The rotational angular velocity of the simulator axis coordinate is converted to the rotational angular velocity of the body coordinate as

(18) $$\left[\begin{array}{c} p\\ q\\ r \end{array}\right]=\left[\begin{array}{ccc} \left(\cos\psi \right) & \left(\cos\phi \right)\left(\cos\psi \right) & 0\\ -\left(\sin\psi \right) & \left(\cos\phi \right)\left(\cos\psi \right) & 0\\ 0 & -\left(\sin\phi \right) & 1 \end{array}\right]\left[\begin{array}{c} \dot{\phi }\\ \dot{\theta }\\ \dot{\psi } \end{array}\right]$$

Then, to calculate the required platform axis coordinates (x,y,z,ϕ,θ,ψ), you need to find the position $\left(p_{x},p_{y},p_{z}\right)$, the x-direction attitude $\vec{n}$, the y-direction attitude $\vec{o}$ and the z-direction attitude $\vec{a}$. So (10) can be written as

(19) RxRz=Ry−1RIB=[(cosψ)−(sinψ)0(cosϕsinψ)(cosϕcosψ)−(sinϕ)(sinϕsinψ)(cosψsinϕ)(cosϕ)]

Observe that Formula (12) contains the x-direction attitude $\vec{n}$, the y-direction attitude $\vec{o}$ and the z-direction attitude $\vec{a}$, we can get

(20) $$\begin{array}{l} \left[\begin{array}{ccc} n_{x}\left(\cos\theta \right)-n_{z}\left(\sin\theta \right) & o_{x}n_{x}\left(\cos\theta \right)-o_{z}\left(\sin\theta \right) & a_{x}\left(\cos\theta \right)-a_{z}\left(\sin\theta \right)\\ n_{y} & o_{y} & a_{y}\\ n_{x}\left(\sin\theta \right)+n_{z}\left(\cos\theta \right) & o_{x}\left(\sin\theta \right)+o_{z}\left(\cos\theta \right) & a_{x}\left(\sin\theta \right)+a_{z}\left(\cos\theta \right) \end{array}\right]\\ =\left[\begin{array}{ccc} \left(\cos\psi \right) & -\left(\sin\psi \right) & 0\\ \left(\cos\phi \right)\left(\sin\psi \right) & \left(\cos\phi \right)\left(\cos\psi \right) & -\left(\sin\phi \right)\\ \left(\sin\phi \right)\left(\sin\psi \right) & \left(\cos\psi \right)\left(\sin\phi \right) & \left(\cos\phi \right) \end{array}\right] \end{array}$$

Then the above formula can be written as

(21) $$a_{x}\left(\cos\theta \right)-a_{z}\left(\sin\theta \right)=0$$

(22) $$\left(\tan\theta \right)=\left(\frac{\sin\theta }{\cos\theta }\right)=\left(\frac{a_{x}}{a_{z}}\right)$$

Take the arc tangent function to get the rotation angle of the pitch axis

(23) $$\theta =\left[\tan^{-1}\left(\frac{a_{x}}{a_{z}}\right)\right]$$

After the θ angle is obtained from Equation (23), it can be listed according to the relationship of Equation (20)

(24) $$\left(\sin\phi \right)=-\left(a_{y}\right)$$

(25) $$\left(\cos\phi \right)=\left[a_{x}\left(\sin\theta \right)+a_{z}\left(\cos\theta \right)\right]$$

(26) $$\left(\sin\psi \right)=\left[-o_{x}n_{x}\left(\cos\theta \right)+o_{z}\left(\sin\theta \right)\right]$$

(27) $$\left(\cos\psi \right)=\left[n_{x}\left(\cos\theta \right)-n_{z}\left(\sin\theta \right)\right]$$

From Equations (24) and (25), the angle of the roll axis can be obtained

(28) $$\left(\tan\phi \right)=\left(\frac{\sin\phi }{\cos\phi }\right)=\left[\frac{-\left(a_{y}\right)}{a_{x}\left(\sin\theta \right)+a_{z}\left(\cos\theta \right)}\right]$$

The yaw axis angle of the outermost frame is

(29) $$\left(\tan\psi \right)=\left(\frac{\sin\psi }{\cos\psi }\right)=\left[\frac{-o_{x}n_{x}\left(\cos\theta \right)+o_{z}\left(\sin\theta \right)}{n_{x}\left(\cos\theta \right)-n_{z}\left(\sin\theta \right)}\right]$$

Taking Equations (28) and (29) as the arc tangent function, we get

(30) $$\phi =\left[\tan^{-1}\frac{-\left(a_{y}\right)}{a_{x}\left(\sin\theta \right)+a_{z}\left(\cos\theta \right)}\right]$$

(31) $$\psi =\left[\tan^{-1}\frac{-o_{x}n_{x}\left(\cos\theta \right)+o_{z}\left(\sin\theta \right)}{n_{x}\left(\cos\theta \right)-n_{z}\left(\sin\theta \right)}\right]$$

The above-mentioned Equations (23), (30) and (31) are used to obtain the coordinates of the three rotating axes of the platform, and the relationship between the surge, sway and heave of the three linear axis coordinates is written by Equation (12), as

(32) $$x=\left(p_{x}\right),\;y=\left(p_{y}\right),\;z=\left(p_{z}\right)$$

The Equations (23) and (32) required to obtain the six axis coordinates are obtained.

## 4. Implementation

**Figure 5:**
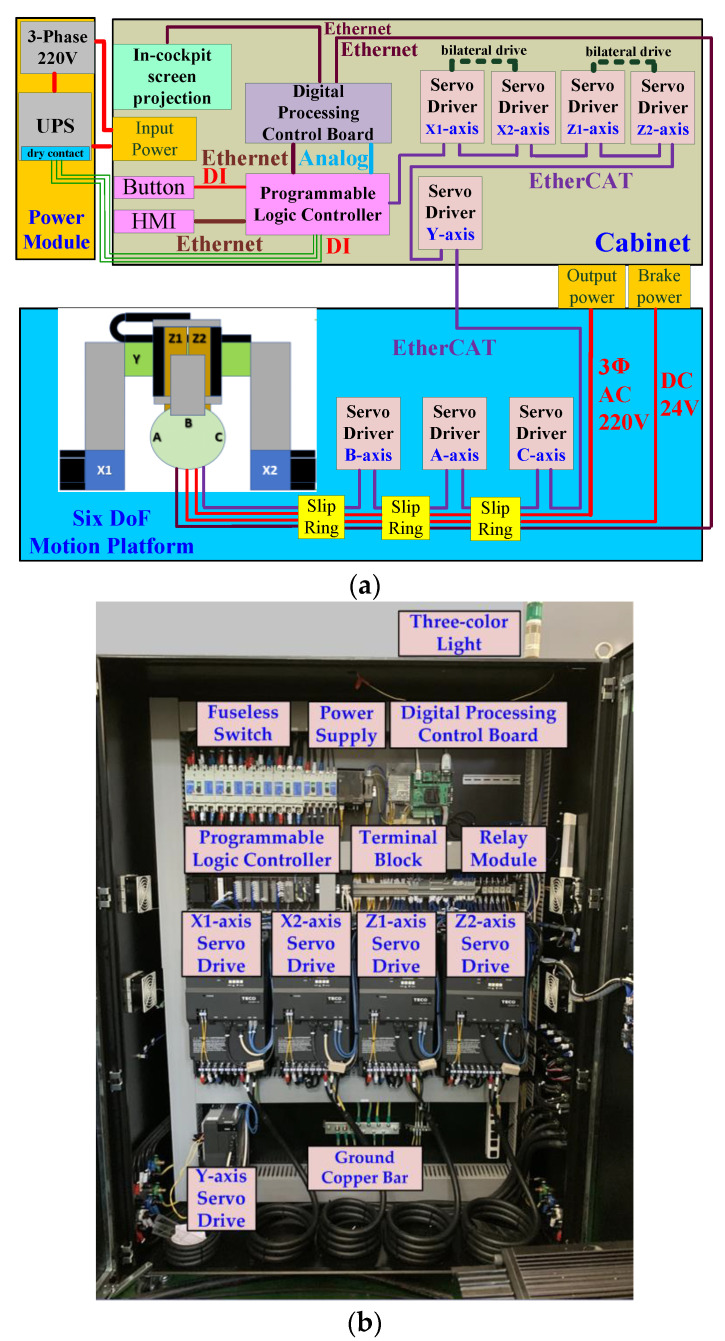
6-DoF motion system: (**a**) control system block diagram; (**b**) cabinet.

**Figure 6:**
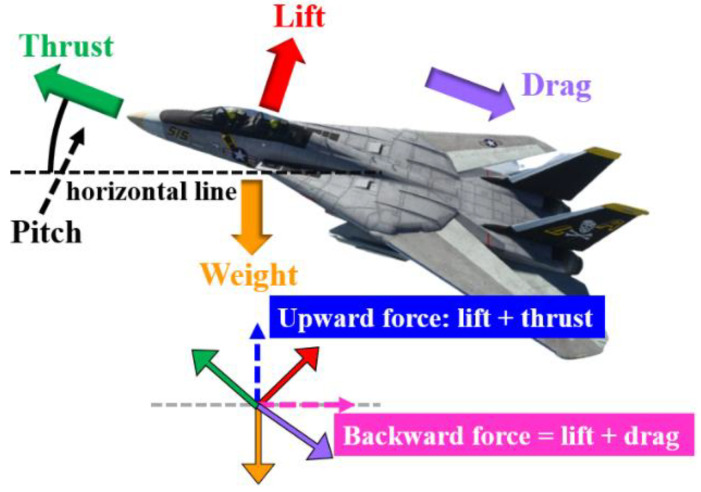
Four forces during takeoff.

**Figure 7:**
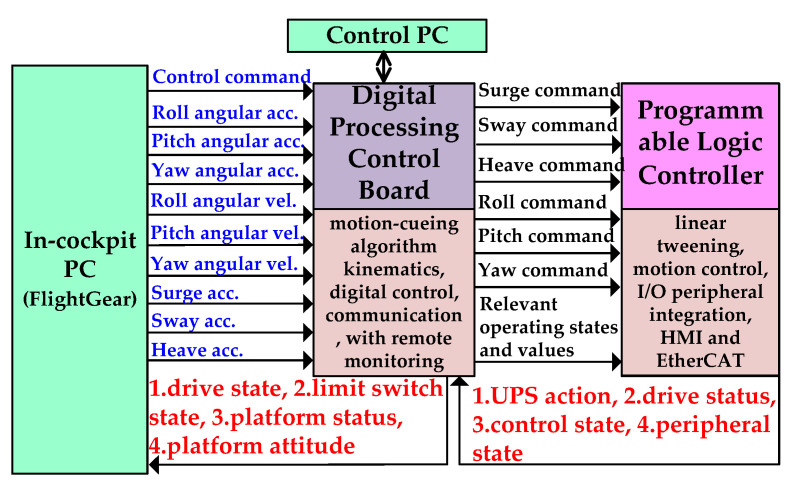
Flight simulator operation procedure diagram.

Since the motion platform proposed in this paper has continuous rotation, it was necessary to install three collector slip rings on the inner frame, outer frame and upper and lower axis slider seats to transmit the power and control signals required for driving the pitch axis, roll axis and yaw axis, respectively, as well as for use inside the cockpit. Figure 5a shows the control system block diagram. The power source is mains or uninterruptible power supply system (UPS). The *A*, *B* and *C*-axes are designed with collector slip rings to achieve unlimited angular rotation. The cabinet shown in Figure 5b is composed of a digital processing control board, a handle, a programmable logic controller, a human-machine interface (HMI), eight servo drives (three sets are installed on the rotating mechanism), and peripheral modules. The digital processing control board uses the digital signal processor (TMS320F28377D) produced by Texas Instruments to write the kinematic model, motion-cueing algorithm, control computer programming interface and visual effect computer transmission interface, and combine the programmable controller NJ-501 reached with HMI. The crew cabin uses FlightGear software to develop the aircraft model to generate the four forces required for flight, as shown in Figure 6. The joystick, pedal and accelerator in the cockpit use the USB interface to read the pilot’s manipulation commands to control the ailerons, rudders and elevators of the aircraft. Figure 7 is the operation procedure. Data is exchanged between the visual effect computer and the digital processing control board, and the platform status, data, driver status and limit status are returned for status control. The detailed comparison is shown in Table 1.

## 5. Experimental Validation

**Figure 8:**
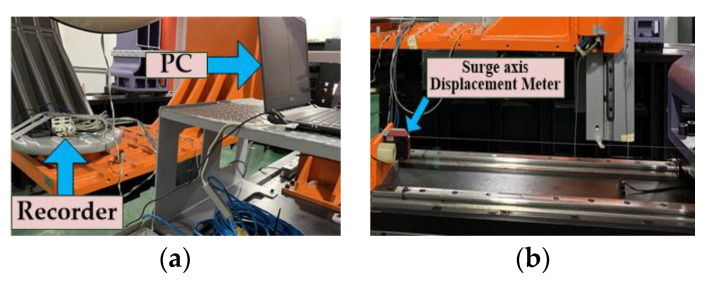

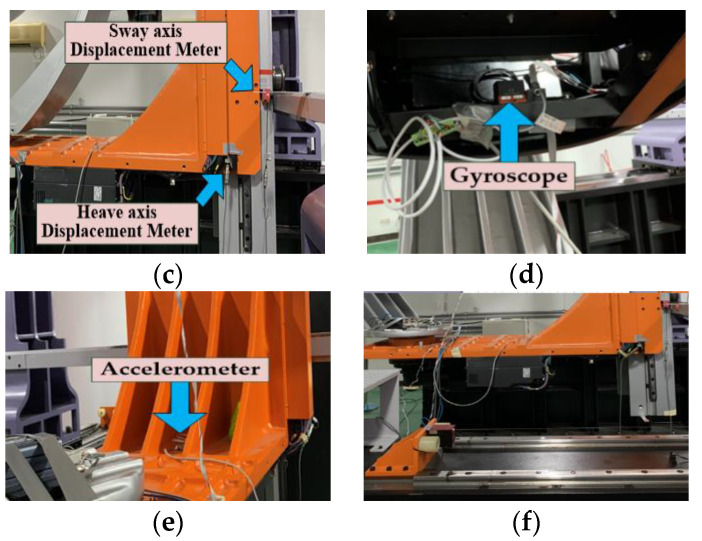
Dynamic performance measurement system: (**a**) recorder installation; (**b**) surge axis displacement gauge installation; (**c**) sway axis and heave axis displacement gauge installation; (**d**) gyroscope installation; (**e**) accelerometer installation; and (**f**) test partial screen.

In order to detect the operating status of the platform, a set of dynamic performance measurement system is built, which is mainly composed of a high-level recorder, three displacement meters, a gyroscope and a three-axis accelerometer. The installation position is shown in Figure 8a–f. The somatosensory response of the pilot in the cockpit was tested, as well as the reading and reception of network packets when the platform moved in different attitudes, the simulation of in-cockpit manipulation and the connection. The instrument Specifications in Table 2. Table 3 shows the measurement results.

**Figure 9:**
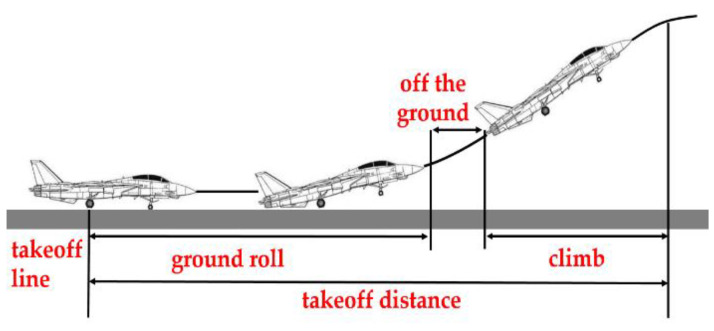
Flight simulator from stationary to take-off action.

The purpose of this experiment is to combine the platform with the visual effect software, and actually test the flight motion effect of the flight simulator combined with the aircraft model. Table 4 organizes the flight simulator verification results of 10 items, and Figure 9 shows the static to takeoff action in the airport runway. Items are as follows:

Item 1: Boarding mode

**Figure 10:**
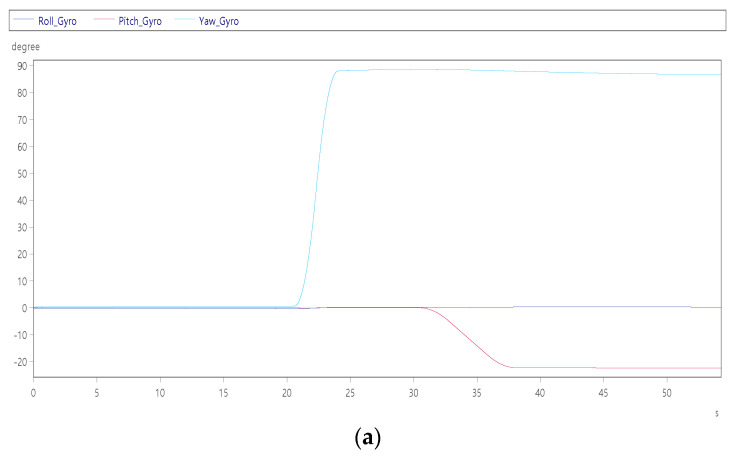

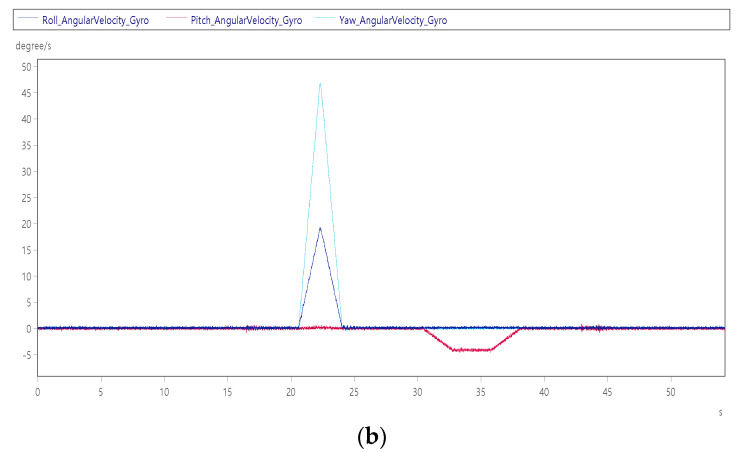
Dynamic response of personnel boarding mode: (**a**) roll, pitch and yaw angles; and (**b**) roll, pitch and yaw angular velocity.

After the origin of the platform is moved to the position of the boarding stairs, in order to make it easier for people to get down, the cockpit will be tilted 22° and the yaw axis will be rotated 90° to let people down. The detailed results are shown in Figure 10a,b. Figure 10a shows three rotation angle attitudes, and the yaw axis is turned to +90° in total from figure. In order to make it safer for people to get out of the cabin, it is known from the figure at 30 s that the pitch axis is slightly downward tilt −22°. Figure 10b shows at 20 s, the yaw axis is seen turning at a maximum speed of +47°/s, and the pitch axis is tilted down at a maximum angular velocity of −4°/s in Figure 10b.

Item 2: Enter the cockpit test

**Figure 11:**
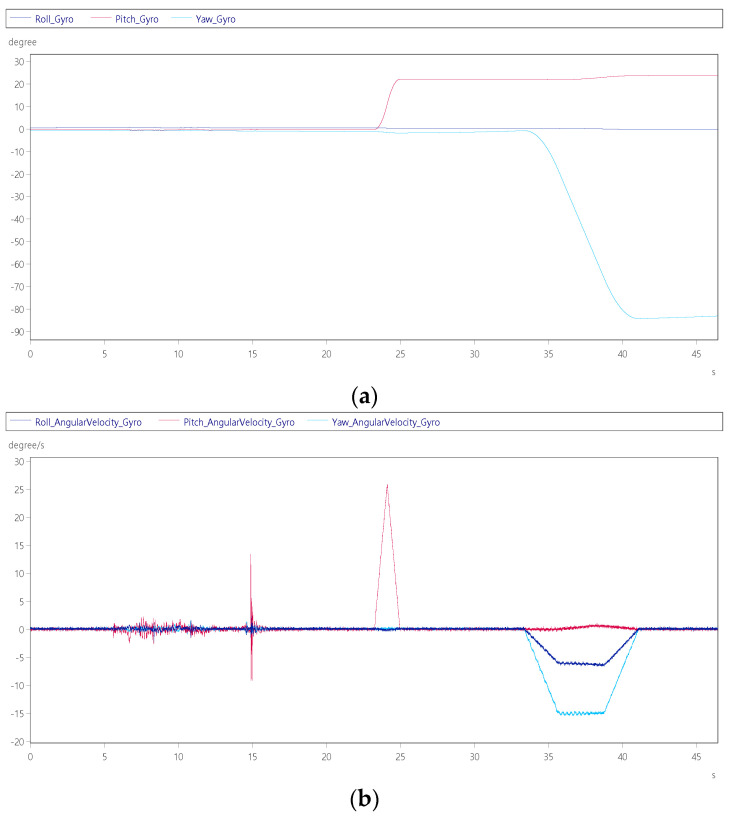
Dynamic response of personnel entering the cockpit: (**a**) roll, pitch and yaw angle; (**b**) roll, pitch and yaw angular velocity.

The pilot enters the cockpit from the boarding ladder and closes the door. Before the cockpit reaches the flight position, it will first tilt up and then start to turn to the flight position. After the person enters and closes the hatch, the pitch axis of the visual effect screen will be tilted up +22° first, as shown in Figure 11a at 23 s. At 40 s in Figure 11a, the yaw axis will eventually turn −90° and adjust to the position where the boarding ladder can connect to the door. Figure 11b shows at 5 s, after the pilot is seated, the cockpit is closed, and a slight shaking signal can be seen in the middle of 15 s, and at 33~41 s, the platform moves from the boarding position to the initial position of the flight take-off, its maximum yaw axis steering speed is –16°/s.

Item 3: Aircraft ground taxi runway test

**Figure 12:**
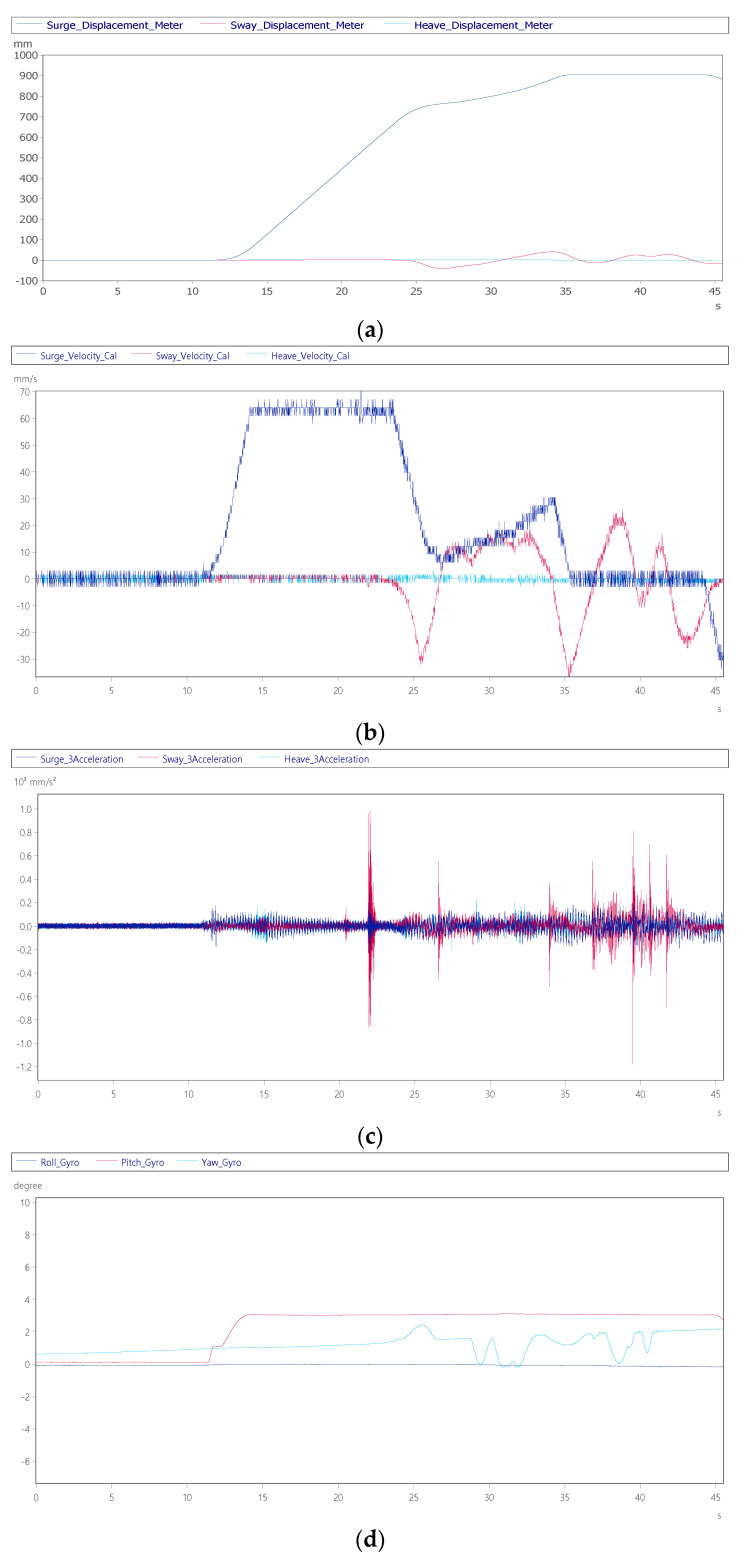

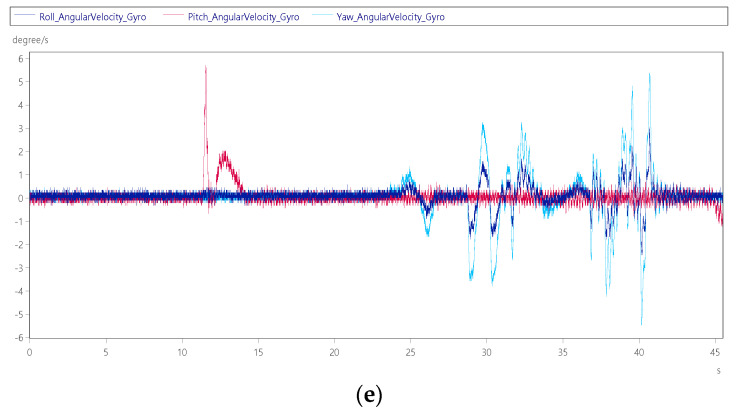
Dynamic response of aircraft ground roll: (**a**) displacement of surge axis, sway axis and heave axis; (**b**) surge axis, sway axis and heave axis velocity; (**c**) surge axis, sway axis and heave axis acceleration; (**d**) roll, pitch and yaw angle; and (**e**) roll, pitch and yaw angular velocity.

When the cockpit moves to the initial take-off simulation position, the pilot in the cockpit can watch the visual software from the screen and control the aircraft. After starting the control, they will taxi on the runway first, and control the joystick, pedal and accelerator. The aircraft is taxiing on the airport runway. During the process, the personnel need to continuously adjust the aircraft to maintain the middle of the runway road surface. Figure 12a shows that the taxiing moves from 0 mm at 12 s to 35 s to the 900 mm position, and the aircraft taxiing speed is matched with the throttle. After lifting, it was observed that within 25~45 s, the sway axis of the nose were continuously adjusted by about ±30 mm to align with its own runway road surface, and the yaw angle attitude was adjusted in Figure 12d. Figure 12b shows that the maximum speed of the surge axis at 15 s is 70 mm/s and the speed of the sway axis is within ±35 mm/s. Figure 12d shows that the during the taxiing process, the pitch axis is raised by 3° to create the aircraft accelerated back feel.

Item 4: Aircraft climb test

**Figure 13:**
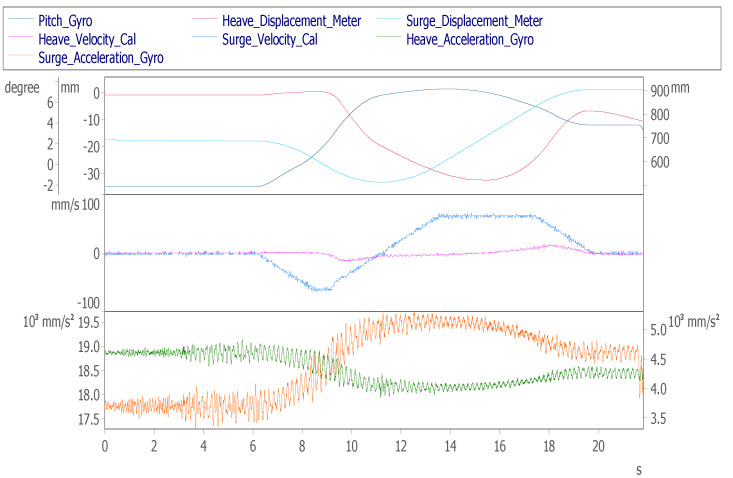
Dynamic response of aircraft lifts off the ground (Top: Attitude, Middle: Velocity, Bottom: Acceleration).

In order to grasp the dynamic effect of the instant take-off of the aircraft, the recorder starts recording in advance when the aircraft taxis on the runway at a speed of 150 km/h, and when the speed reaches 200 km/h, the nose of the aircraft is pulled up to take off, which corresponds to 6 s in Figure 13 (top) place. After 14 s, the aircraft will continue to fly in level flight after reaching a certain altitude and stop recording after about 10 s. Operate the visual effect software. When the speed of the aircraft reaches 150 km/h, the recorder starts to record. When the speed reaches 200 km/h, pull the nose of the aircraft. It can be seen from Figure 13 (bottom) shows that the pitch axis is from −2° to +6°. The total elevation angle is about 8°, and the surge axis reach the maximum speed of +70 mm/s at 14 s after the aircraft is pulled up and taken off.

Item 5: Aircraft roll left and right roll test

**Figure 14:**
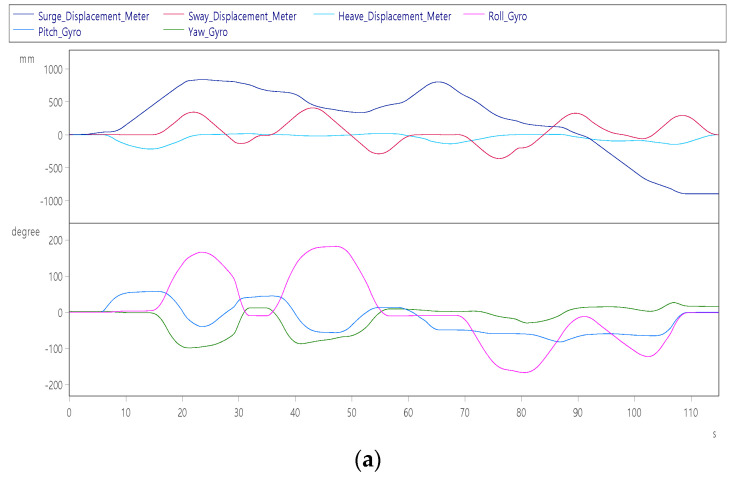

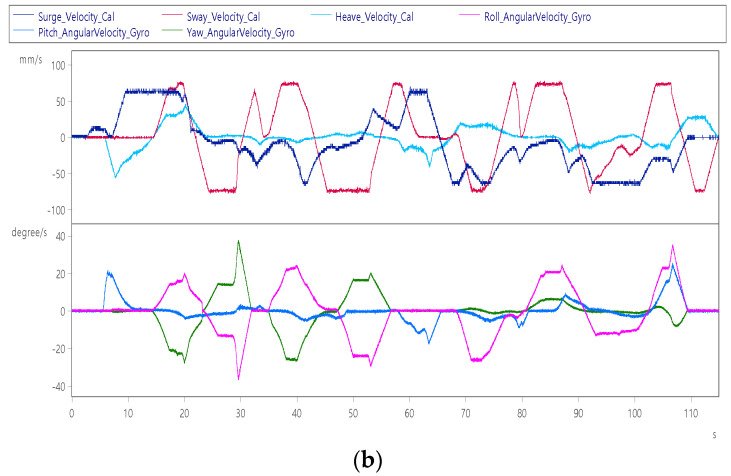
Dynamic response of aircraft roll left and right: (**a**) displacement (up) and angle (down); (**b**) velocity (up) and angular velocity (down).

After a period of level flight, the subjects of continuation item 4 continued to control the aircraft to perform left and right rolls, and after 10 s from the start, they performed two consecutive right rolls while pulling the nose +60°, as shown in Figure 14a. It can be seen from the figure that the maximum surge axis position of the platform is +1000 mm. After maintaining the normal flight attitude for 55~70 s, then at 70 s, press the nose to dive while performing two consecutive left rolls. It can be seen that the platform will retreat to the −1000 mm position. Figure 14b shows that the left and right rolls are performed with a maximum roll angular velocity of ±30°/s, a maximum pitch angular velocity of ±20°/s and a maximum yaw angular velocity of +35°/s complete two rolls each in about 110 s.

Item 6: Aircraft hovering test

**Figure 15:**
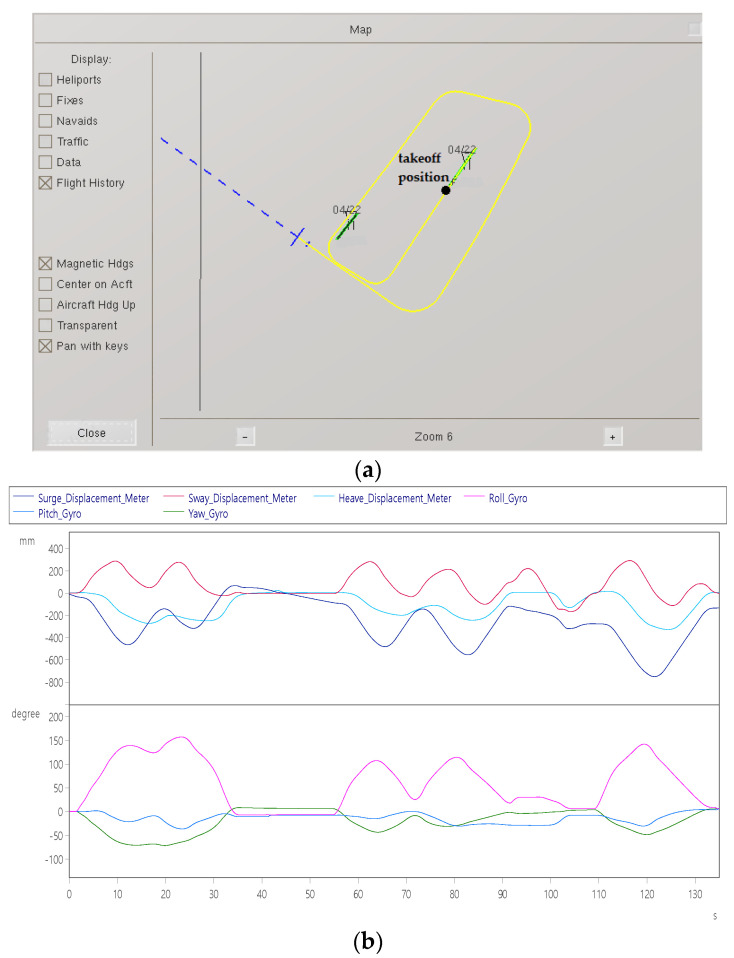

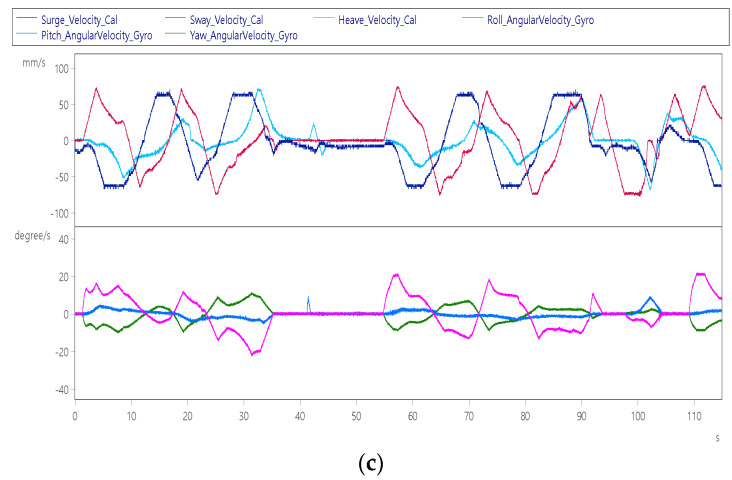
Hover dynamic response: (**a**) aircraft circling trajectory; (**b**) displacement (up) and angle (down); and (**c**) velocity (top) and angular velocity (bottom).

Figure 15a shows the visual effect software taking off from the airport runway and starting to circle around the airport to the right. Figure 15b shows that the bottom picture shows that the first turn is performed after 2 s, and it circles along the airport. The maximum yaw angle is −90°, and the maximum roll angle is about +180°. The second turn is performed at 55 s, with a yaw angle of −40° and a maximum roll angle of approximately +120°. And perform the third turn at 100 s. At this time, the yaw angle is −45°, and the maximum roll angle is about +180°. Figure 15c shows that the maximum roll angular velocity is ±20°/s.

Item 7: Aircraft inverted flight test

**Figure 16:**
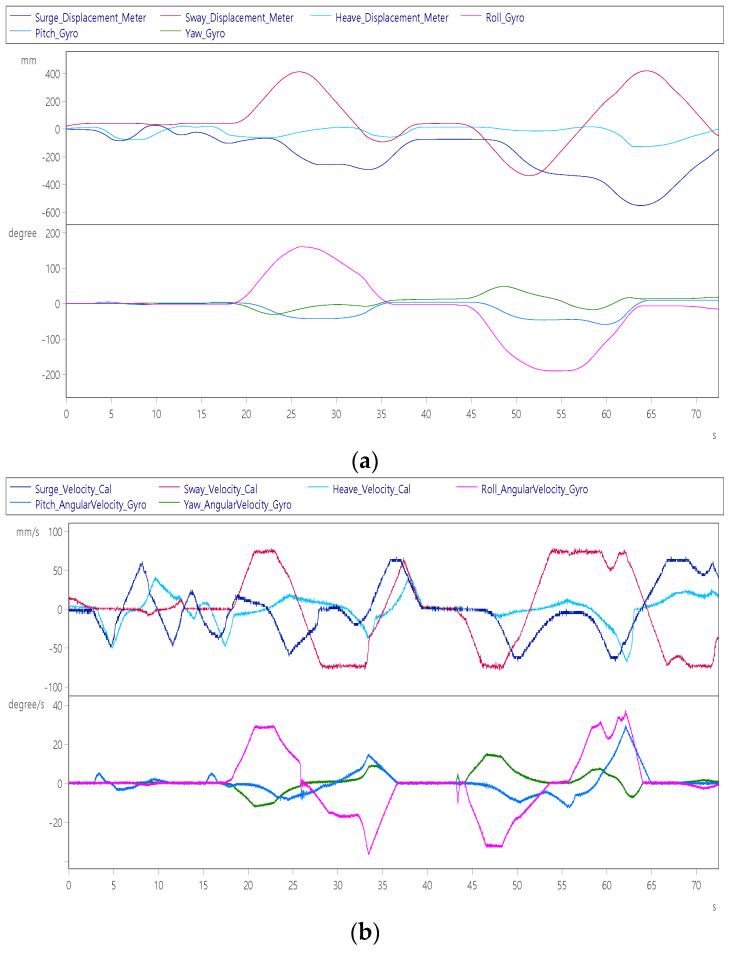

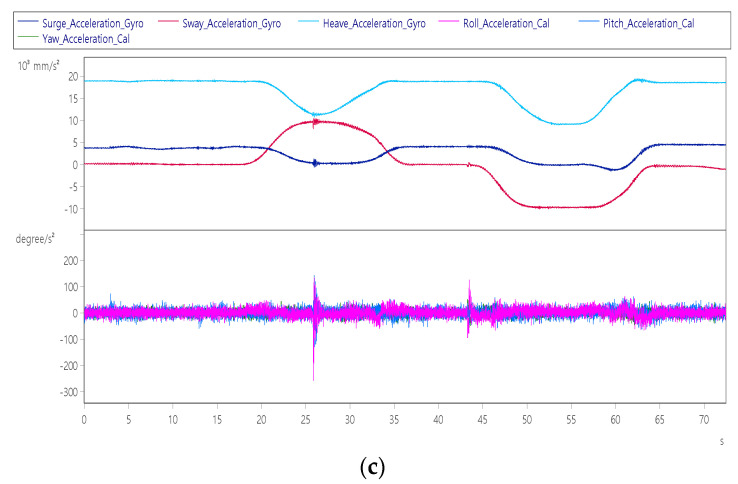
Dynamic response of the aircraft in inverted flight: (**a**) displacement (up) and angle (down); (**b**) velocity (up) and angular velocity (down); (**c**) acceleration (up) and angular acceleration (down).

In order to understand the performance of the platform movement when the aircraft is flying upside down, first perform a right roll to +180° and then fly backwards for 10 s, then turn right for about 10 s, then switch to the left direction and fly backwards for 10 s. From the bottom of Figure 16a, we can clearly see the result of inverted flight after two rolls in different directions. Figure 16b shows that the first roll angular velocity reaches ±32°/s, the second roll angular velocity reaches ±34°/s, and the flight direction is turned right at 64 s.

Item 8: Aircraft landing test

**Figure 17:**
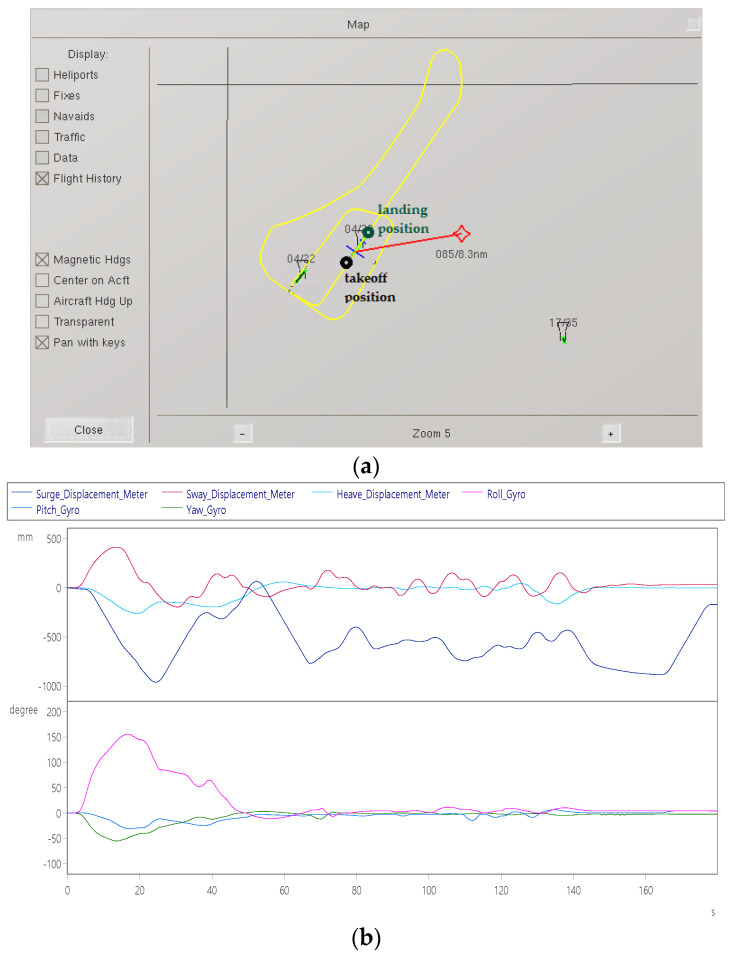

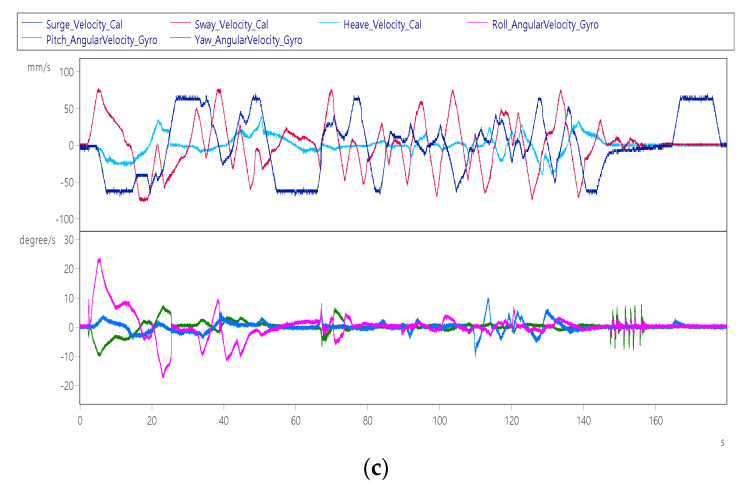
Dynamic response of aircraft landing: (**a**) landing trajectory after hovering; (**b**) displacement (up) and angle (down); and (**c**) velocity (up) and angular velocity (down).

The purpose of the test is to simulate the landing of the aircraft in the air through the visual effect software. When the aircraft is hovering at a high altitude, it can be seen from Figure 17a that after performing a right roll at the outermost strip. Figure 17b shows that after aligning with the runway, the aircraft will begin to lower altitude around 70 s. When approaching the airport, the landing gear will be lowered. At 115 s, the pitch angle is about −15° and touches the ground (Figure 17b). The angular velocity is about ±10°/s movement (Figure 17c).

Item 9: Aircraft re-flying test

**Figure 18:**
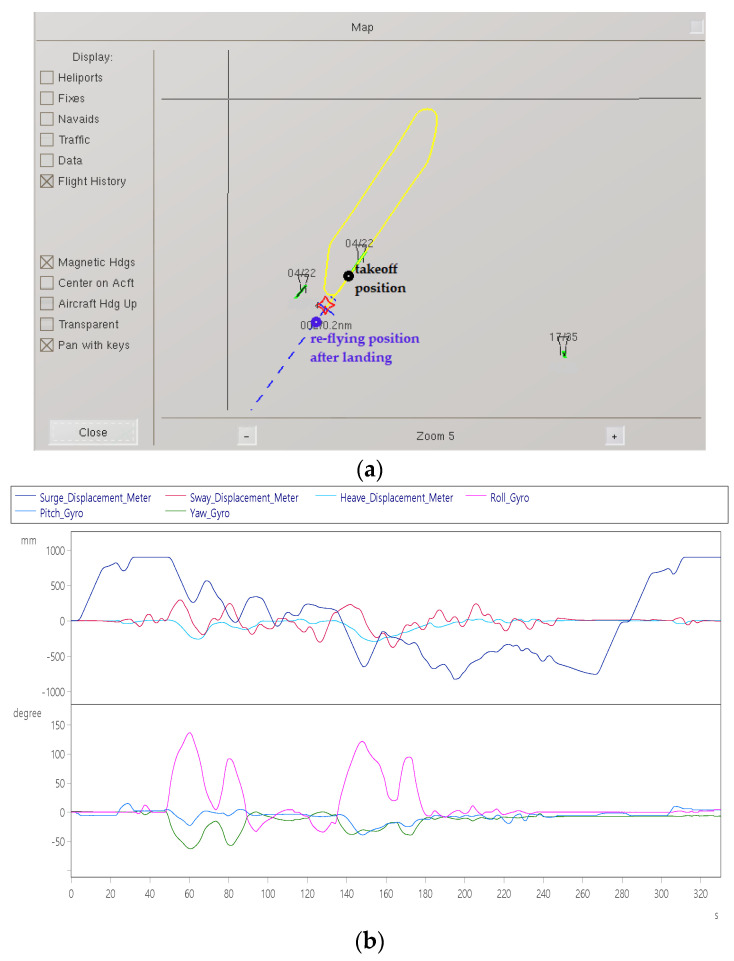

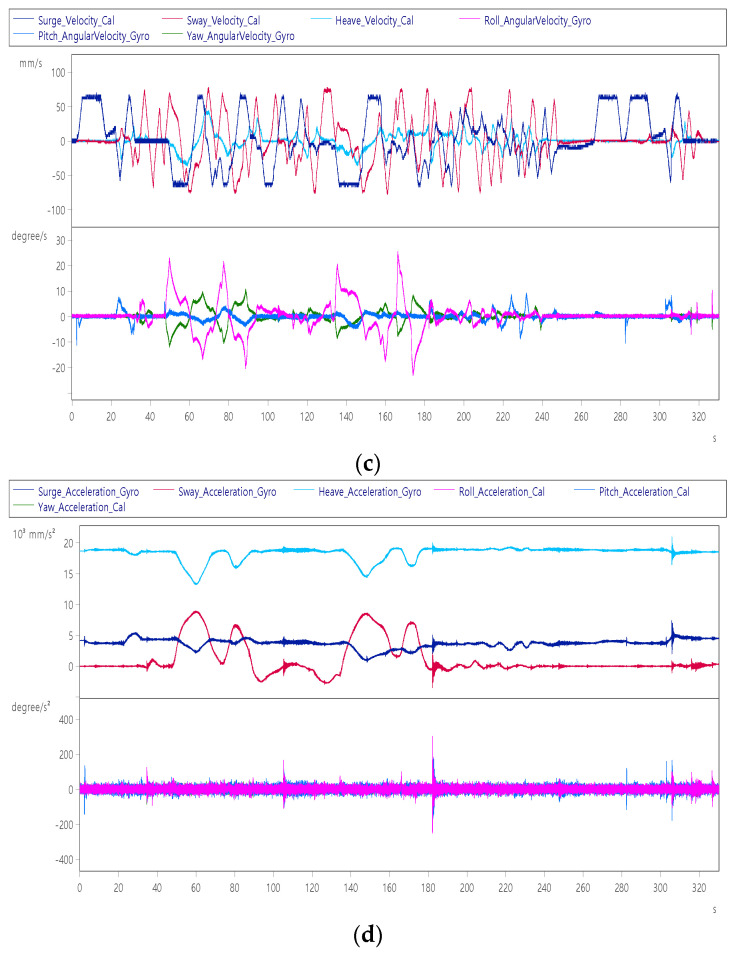
Dynamic response of aircraft re-flying: (**a**) take-off trajectory after landing; (**b**) displacement (up) and angle (down); (**c**) velocity (top) and angular velocity (bottom); and (**d**) acceleration (top) and angular acceleration (bottom).

The pilot cooperates with the visual effect software (Figure 18a) to execute the take-off of the aircraft. Figure 18b shows that the platform starts to move +900 mm before and after take-off at 5 s, and at 25 s, the nose is pulled up and the lift-off angle is +20°. Start the first turn at 50 s with a roll angle of +150° and a roll angle of +25°/s (Figure 18c). For the second turn, the roll angle is +130°, and the speed of the turn and roll angle is +25°/s (Figure 18c). After completion, it will fly straight to the runway and land. The nose tilted down −15° and then contacted the runway and then started braking until it came to a complete stop at 255 s, waited for 10 s, and performed a re-fly test at 265 s. As shown in Figure 18b, it started to taxi again, and the aircraft was pulled up at 305 s. Take off at a head depression angle of about +8°, and start level flight for 10 s after takeoff to stop the record.

Item 10: Leave cockpit mode

**Figure 19:**
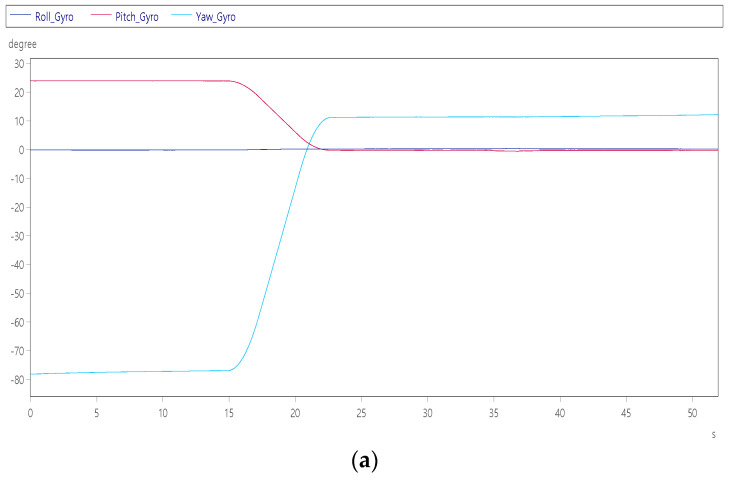

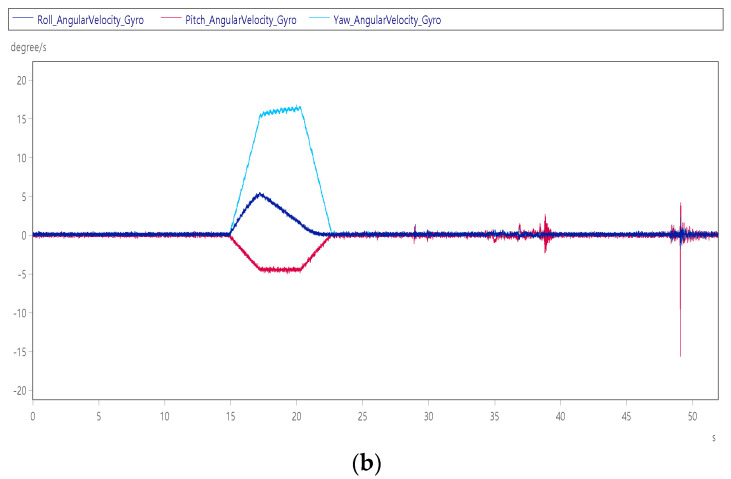
Dynamic response of people leaving the cockpit: (**a**) three-axis angle; and (**b**) three-axis angular velocity.

When items 1 to 9 are completed, the test personnel in the cockpit will stay in the final position with a roll angle of 0°, a pitch angle of +22.5°, and a yaw angle of −77.5°. It can leave the position, which is convenient for people to get down from the boarding ladder. Figure 19a shows that the platform moves to the roll angle of 0°, the pitch angle of 0° and the yaw angle of +10° at 15 s for people to get off. Figure 19b shows that the yaw axis is turning at a maximum speed of +17°/s, with a total turning of +90° and a slight jitter can be clearly seen at 34~38 s, indicating that the pilot is moving from the cockpit at this time leave within.

## 6. Conclusions

This paper proposes the design, implementation and measurement of a digital control system. By integrating the visual effect software FlightGear, a control joystick, a pedal, an accelerator, a flight simulator is actually built, and the motion-cueing algorithm is applied to the motion platform for flight testing to provide pilots with a real-time, safe, efficient, and risk-free simulated aircraft, and training equipment that can perform different flight actions during the flight. In addition to the stroke closed loop used by the simulator, the control mode can also be used in the adjustable speed drive closed loop. Through the digital architecture proposed in this paper, the information transmission and control between the platform and the cockpit can be achieved, and the lengthy calculation time of the previous simulators using the motion control card can be solved. The relevant items are carried out by the pilot and the cockpit in the simulated driving of the aircraft, and the actual verification of the proposed new motion platform for six-axis flight training achieves good experimental results, including the long-distance stroke, continuous rotation, and other related actions.

## Figures and Tables

**Table 1:** The comparison of different flight simulator.

	Proposed System	Teufel et al. [25]	Pan et al. [26]
Motion-cueing algorithm	Yes	Yes	No
Real-time capable	High	Medium	Medium
Scalability	High	High	High
Implementation complexity	Medium	Medium	Low
Power dissipation	84.5 kW	41 kW	14.25 kW
Supporting weight of platform	>400 kg	500 kg	400 kg
Realization	Embedded System	PC-based	PC-based
Number of axes	6	6	4
Communication	EtherCAT, Ethernet, Analog and Digital Communication	CAN bus and Ethernet	EtherCAT

**Table 2:** Instrument specifications.

Item	Type	Specification	Number
Recorder	imc CS 5008	Measuring strain signal ± 1000 mV/V… ±5 mV/V. Measure the voltage ± 10 V… ±5 mV. Support CAN Bus Protocol.	1
Long distance displacement meter	DP- 2000G	Measuring range ± 1 m. Output signal 5 mV/V ± 0.3%. Working voltage 10 V.	1
Medium distance displacement meter	DP- 1000G	Measuring range ± 0.5 m. Output signal 5 mV/V ± 0.3%. Working voltage 10 V.	2
Gyroscope	MTi- 680G	Attitude angle Yaw & Roll ± 180°, Pitch ± 90°. Acceleration range ± 10 g. Angular velocity range ± 2000°/s.	1
Accelerometer	2406- 025	Measure X, Y, Z axes. Measuring range ± 25 g.	1

**Table 3:** The measurement results.

DoF	Stroke	Velocity	Acceleration
Surge	±1000 mm	>400 mm/s	>4000 mm/s^2^
Sway	±500 mm	>400 mm/s	>4000 mm/s^2^
Heave	±500 mm	>400 mm/s	>4000 mm/s^2^
Roll	360°	>50°/s	>50°/s^2^
Pitch	360°	>100°/s	>100°/s^2^
Yaw	360°	>100°/s	>100°/s^2^

**Table 4:** Description of test items.

Item	Description	Recorder Display Channel	Maximum Dynamic Value	Figure
Boarding mode	The pilot waits for the cockpit to move to the boarding point.	Yaw axis Angle	+90°	Figure 10a
		Pitch axis Angle	−22°	
		Yaw angular velocity	+47°/s	Figure 10b
		Pitch axis angular velocity	−5°/s	
Enter the cockpit	The pilot opens the door to enter from the boarding point, and then closes the door.	Yaw axis Angle	−85°	Figure 11a
		Pitch axis Angle	+22°	
		Yaw axis angular velocity	−15°/s	Figure 11b
		Pitch axis angular velocity	+25°/s	
Aircraft ground taxi runway	The pilot controls the flight simulator to taxi on the runway.	Surge axis displacement	+900 mm	Figure 12a
		Sway axis displacement	±30 mm	
		Surge axis speed	+65 mm/s	Figure 12b
		Sway axis speed	±35 mm/s	
		Pitch axis Angle	+3°	Figure 12d
Aircraft climb	The pilot accelerate the aircraft to 200 km for climbing.	Pitch axis Angle	+9°	Figure 13
		Sway axis displacement	−30 mm	
		Surge axis displacement	+400 mm	
Aircraft roll left and right roll	The pilot controls the aircraft roll left twice and roll right twice in a row.	Roll axis Angle	±180°	Figure 14a
		Sway axis displacement	±300 mm	
		Surge axis speed	±60 mm/s	Figure 14b
		Sway axis speed	±70 mm/s	
Aircraft hovering	Pilot controlled aircraft hovering in the air.	Surge axis displacement	−700 mm	Figure 15b
		Sway axis displacement	+280 mm	
		Surge axis speed	±60 mm/s	Figure 15c
		Sway axis speed	±70 mm/s	
Aircraft inverted flight	The pilot controls the flight to roll 180° to the left and to the right.	Roll axis angle	±180°	Figure 16a
		Sway axis displacement	±380 mm	
		Roll axis speed	±35°/s	Figure 16b
		Sway axis speed	±75 mm/s	
		Sway axis acceleration	±900 mm^2^	Figure 16c
Aircraft landing	Pilots steer aircraft for landing.	Surge axis displacement	−1000 mm	Figure 17b
		Sway axis displacement	+480 mm	
		Roll axis Angle	+170°	
		Yaw axis Angle	−60°	
		Surge axis speed	±60 mm/s	Figure 17c
		Sway axis speed	±70 mm/s	
		Roll axis speed	±22°/s	
Aircraft re-flying	Pilot re-flying after landing.	Surge axis displacement	±900 mm	Figure 18b
		Roll axis Angle	+145°	
		Yaw axis Angle	−50°	
		Pitch axis Angle	±15°	
		Surge axis speed	±60 mm/s	Figure 18c
		Sway axis speed	±70 mm/s	
		Roll axis speed	±22°/s	
		Yaw axis angular velocity	±10°/s	
Leave cockpit mode	The pilot completes the mission and enters exit cockpit mode.	Yaw axis Angle	+90°	Figure 19a
		Pitch axis Angle	−22°	
		Yaw axis angular velocity	+17°/s	Figure 19b

## Data Availability

This study did not report any data.
